# India Ink Tattooing of Ureteroenteric Anastomoses

**DOI:** 10.3390/tomography9020037

**Published:** 2023-02-21

**Authors:** Mei N. E. Tuong, Grace E. Prillaman, Stephen H. Culp, Marc Nelson, Tracey L. Krupski, Sumit Isharwal

**Affiliations:** 1 Department of Urology, University of Virginia Health System, Charlottesville, VA 22903, USA; 2 School of Medicine, University of Virginia, Charlottesville, VA 22903, USA; 3 Uropartners, Glenview Illinois, Chicago, IL 60007, USA

**Keywords:** India Ink, ureteroenteric anastomosis, urinary diversion, ureteroenteric anastomotic strictures, post-anastomotic imaging

## Abstract

While upper tract access through the insensate conduit following urinary diversion takes less time and incurs fewer costs than percutaneous kidney access does for the treatment of ureter and kidney pathology, endoscopic ureteroenteric anastomoses (UEA) identification can be difficult. We injected India Ink into the bowel mucosa near the UEA during ileal conduit diversion (IC) to determine the safety and feasibility of ink tattooing. Patients undergoing IC were prospectively randomized to receive ink or normal saline (NS) injections. The injections were placed 1 cm from UEA in a triangular configuration, and loopogram exams and looposcopy were performed to identify reflux (UR), UEA, the tattooing site and strictures in 10 and 11 patients randomized with respect to ink and NS injections, respectively. Ink patients were older (72 vs. 61 years old, *p* = 0.04) and had a higher Charlson Comorbidity Index (5 vs. 2, *p* = 0.01). Looposcopy was performed in three ink and four NS patients. Visualization of UEA was achieved in 100% of the ink and 75% of the NS patients (*p* = 0.26). The ink ureteroenteric anastomotic stricture (UEAS) rate was higher (*N* = 3 vs. *N* = 1) and six patients vs. one patients underwent surgery, respectively, for UEAS (*p* = 0.31). The study was halted early due to safety concerns. Our pilot study demonstrates that ink can be well visualized following injection near UEA during IC. However, the ink cohort had more UEAS than previously cited in the literature and our prior institutional UEAS rate of 6%. While this study sample is small, the higher incidence of UEAS after ink injection led us to question the utility and safety of ink injection following IC.

## 1. Introduction

Incontinent urinary diversions are employed in cases that are malignant and benign and are created in either the small intestine or large bowel. The ileum is the most common tissue used due to more favorable metabolic aberrations and its ease of preparation. Ureteroenteric diversions are at risk of numerous complications that can affect the upper urinary tract system; this may require the endoscopic identification of the ureteroenteric anastomotic site (UEA), which can be difficult [1].

Stone disease is the most common indication for an upper tract procedure following urinary diversion. In a recent study looking at 54 retrograde ureteroscopy procedures in patients with an ileal conduit, the most common indication for an upper tract procedure was stone disease (an incidence rate of 35.2%) [2]. Other indications included the diagnosis of upper urinary tract malignancies, the need for diagnostic flexible ureteroscopy, the removal of encrusted stent/nephrostomy tube, and urine leaking after the diversion. The successful cannulation of the ureter from within the ileal conduit occurred in 74% of cases, but 24% of retrograde flexible ureteroscopies need simultaneous percutaneous antegrade access to the upper urinary tract system. Of the cases with no access to the ureter, the majority were due to the inability to locate the ureter in a tortuous ileal conduit or due to the inability to identify the ureters or ureteral orifice. In another study, a similar success rate of 75% was reported in ileal conduits, and the majority of ureteral access failures were also due to a tortuous ileal conduit [3].

Another serious complication of an ureteroenteric diversion is the development of an UEA stricture, which predisposes a patient to increased risk of pyelonephritis, obstructive uropathy, and renal calculi. The incidence of developing renal damage secondary to obstructive uropathy after undergoing an ileal conduit (IC) has been reported to be as high as 11% 15 February 2023 9:19:00 P.M. The rate of UEA stricture occurrence is estimated to range from 0–11.1% per patient and up to 4.5% per anastomosis [4,5,6,7,8].

A UEA stricture may be treated endoscopically or with open surgery. Open revision is the gold standard, with long term success rates of up to 90% [9,10,11,12,13]. However, open revision comes with significant risks to the patient. As a result, minimally invasive endourological approaches are attempted prior to open revision. These endourologic procedures include permanent ureteral stent placement, balloon dilation of the stricture, endoureterotomy using a cold knife or laser, and electrocautery. Endoscopic management often fails due to the inability to locate the ureter in the ureteroenteric diversion, as discussed above, or recurrent structuring due to poorly perfused tissue [2,3].

India Ink (ink) has historically been used during gastrointestinal surgeries to mark specific landmarks [14,15]. Due to the success of ink tattooing in gastrointestinal surgery, there has been interest in using tattoo ink tattoo in urologic procedures. Shatz et al. looked at the long-term safety profile of India Ink tattoos in the colon in 55 tattoo sites [14]. India ink tattoos remained in these patients for 1.5–117 months. There were no clinical complications such as an infection, a fever, or abdominal pain; there were also no endoscopic abnormalities on or adjacent to the tattoo sites and no neoplastic changes in the mucosa overlying the tattoo. Similarly, Shaffer et al. used India Ink to tattoo the squamocolumnar junction of the esophagus and followed 19 patients endoscopically for 36 months [15]. One hundred percent of the patients who remained in the study after 36 months had a good or excellent tattoo persistence without complications related to the India Ink tattooing; there was no chest pain, bleeding, perforation, ulcers, inflammation, pain, or tears in the mucosa.

Kim et al. [16] describes the successful use of ink to mark pathologic bladder lesions in ten patients prior to undergoing laparoscopic partial cystectomy. There has only been one previous study using India Ink to tattoo ureteroenteric anastomotic sites for identification by McCormick et al. [17], who used the ink to mark the UEA while creating urinary diversions. Ink tattooing was performed in five patients undergoing radical cystectomy with IC. During the follow up, these patients underwent flexible looposcopy of the IC with a visualization of the ink tattoo in all patients. No patients experienced any adverse events related to the tattoo, and the tattoo persisted throughout the follow up.

In this study, we hypothesized that the ink tattooing of the UEA would increase UEA identification for future upper urinary tract access. In addition, we sought to assess the safety of ink tattooing of the UEA.

## 2. Materials and Methods

### 2.1. Study Population

Internal review board approval was obtained (IRB #21312) to conduct the study. The patients were both male and female aged from 18 to 80 years old, who were undergoing an open incontinent urinary diversion (either for benign or malignant reasons) by any surgeon within the urology department. Once informed consent was obtained, the patients were prospectively randomized to either receiving injections of ink or the control, 0.9% normal saline. Block randomization was pre-performed using a 1:1 randomization scheme, with a block size of four. Patients and investigators were not blinded to the randomization of ink or the control groups. Exclusion criteria were patients undergoing orthotopic urinary diversion, pregnant women, prisoners, or cognitively impaired patients.

### 2.2. Technique

Spot^®^ Ex Endoscopic Tattoos (Mechanicsburg, PA, USA) were given to patients randomized into the ink group. During the time of creation of a refluxing UEA, three injections of 0.5 mL of either ink or 0.9% normal saline were injected about 1 cm away from the UEA in a triangular fashion. The injections were administered to the submucosal layer of the enteric segment. The type of stent (8F feeding tube or single J stent) and the duration of stent usage was at the surgeon’s discretion. Cystectomy was robotic assisted or it was performed in an open manner, but the diversion was performed in an open fashion. All anastomoses were completed in Bricker fashion. Postoperatively, the patients returned to the clinic for standard of care routine appointments at one, six, and twelve months. Gravity loopogram tests of their urinary diversion were performed at one month to assess for patency of the UEA via reflux in contrast into the upper urinary tract. Gravity loopogram is the standard post-operative protocol used to evaluate strictures. If there was no reflux of either one or both anastomoses, we recommended the patient undergo a clinic looposcopy of the urinary diversion. Looposcopy was performed as part of the study protocol using a flexible 17 French cystoscope to attempt visualization of one or both UEA, assess the presence of India Ink, and if possible, observe efflux from the anastomosis.

### 2.3. Data Collection

Demographic data collected on each patient included age, gender, race, Charlson Comorbidity Index (CCI), and the reason for urinary diversion. Surgical data were also collected: preoperative and postoperative pathology and cancer staging if applicable, preoperative laboratory values, surgical date, and randomization to either the ink or control groups. Postoperative data included the length of the hospital stay, in hospital complications, date of ureteroenteric stent removal, renal function as determined by Glomerular Filtration Rate (GFR), any related postoperative imaging, loopogram date and results, and looposcopy date and results. All patients were followed for one year after their surgery for any complications and additional surgeries or procedures.

### 2.4. Statistical Analysis

Data were collected and stored in our institutional REDCap (Nashville, TN, USA) database. Our primary outcome was to determine the success rate and feasibility of identifying both UEAs during looposcopy. Secondary outcomes were assessing the safety of injecting ink near the UEA. Data are presented as medians with interquartile ranges. Demographic data, surgical outcomes, and complications were compared using Fisher’s exact test or Wilcoxon signed rank sum test, as appropriate. Looposcopy success rate was calculated with Fisher’s exact test. For all of the tests, *p* < 0.05 was considered to be statistically significant. Statistical data analysis was performed in RStudio Version 1.3.1093 (Boston, MA, USA).

## 3. Results

### 3.1. Demographic Data

Overall, there were 10 patients in the ink group and 11 in the control group. Preoperative demographic data are shown in Table 1. Control patients were significantly younger than the ink patients were (median 61 vs. 72 years old, *p* = 0.04). All of the control patients and 90% of the ink patients underwent surgery for malignant conditions, while only one ink patient underwent surgery for a benign pathology. The ink patients had significantly more comorbidities based on the CCI compared with those of the control patients (five vs. two, *p* = 0.01). There was no difference in median preoperative GFRs between the two groups (Ink 65 vs. control 64, *p* = 0.81).

### 3.2. Surgical and Postoperative Demographics

There were no differences between Ink and control patients in type of cystectomy performed, length of hospital stay, and surgical pathology as shown in Table 2. Additionally, there were no differences in hospital complications based on Clavien Dindo score between the two groups (*p* = 0.29). Similarly, the number of patients discharged with ureteral stents in place was not statistically significantly different between the ink and control groups (*p* = 0.64). When we were assessing the GFR 60 and 90 days after surgery, there was no difference between the ink and control cohorts (44 vs. 57, *p* = 0.43; 47 vs. 60, *p* = 0.13, respectively).

### 3.3. Loopogram and Looposcopy

**Figure 1:**
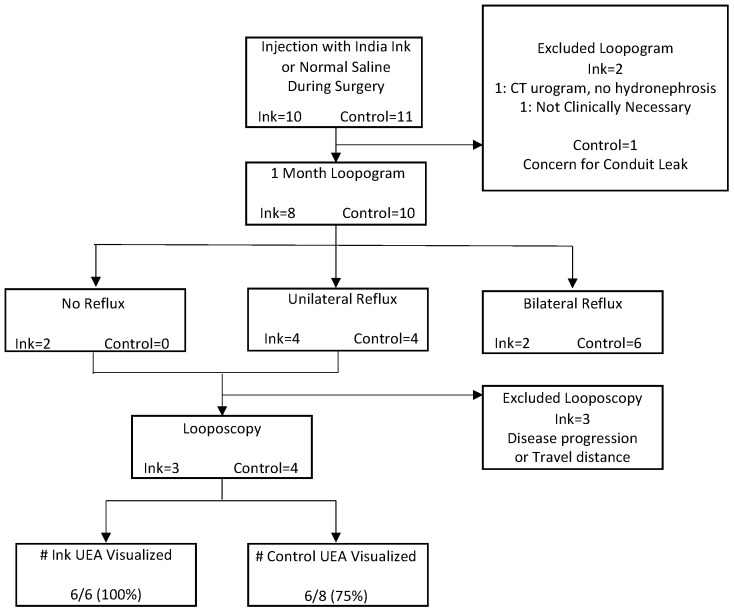
Outcomes of loopogram and looposcopy after India Ink or normal saline injection. Data are presented as number of patients unless stated otherwise. India Ink, Ink; Ureteroenteric Anastomoses, UEA.

**Figure 2:**
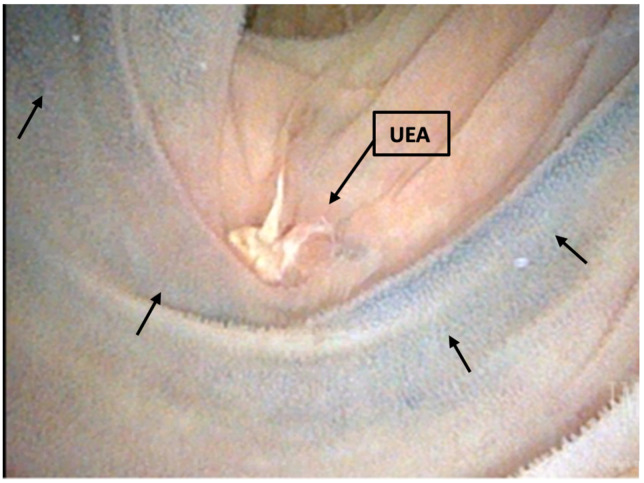
Image of India Ink tattoo at ureteroenteric anastomoses site. Arrows indicate ink tattoos.

Figure 1 describes our loopogram and looposcopy protocol and results. The loopogram tests were performed for 80% (*N* = 8/10) of ink and 91% (*N* = 10/11) of control patients. There was no difference in the number of median days at which the loopogram was performed after surgery or the median time after ureteral stent removal between the ink and control patients (27 days IQR 24–34 vs. 32 days IQR 27–33, *p* = 0.53; 21 days IQR 13–29 vs. 25 days IQR 19–29, *p* = 0.72, respectively). For the patients who had no reflux due to UEA or just unilateral reflux demonstrated by the loopogram, looposcopy was performed in 50% (*N* = 3/6) of the ink and 100% (*N* = 4/4) of control patients. There also was no difference in the number of days after surgery when looposcopy was performed between the ink and control patients (41 vs. 64 days, *p* = 0.06). All UEA (100%) marked with ink were visualized compared to 75% of the UEA marked with the control, although this was not statistically significant (*p* = 0.26) (Figure 2).

### 3.4. Postoperative Complications and Additional Surgeries

Any postoperative complications, beyond initial surgical hospitalization, occurring within the first year after surgery are described in Table 3. Overall, there were 80% and 82% complication rates for the ink and control patients, respectively. There was no difference however between the classification of complications by Clavien–Dindo grading between the two groups (*p* = 0.16). Most complications were related to an infection. Other complications included metastasis leading to death, failure to thrive, incisional hernia, and wound dehiscence.

After the initial urinary diversion, six surgeries in three ink patients compared to one surgery in one control patient were required for the management of a UEA stricture (Table 4). There was no significant difference in the number of surgeries between the two groups (*p* = 0.31). Only one patient in the ink cohort required a ureteral reimplant into the urinary diversion, while most strictures were treated successfully with either a percutaneous nephrostomy tube or ureteral stent. During the open surgical revision, inspection of the intestinal mucosa suggested long-term edema and disruption of the tissue integrity.

## 4. Discussion

Our study supported our hypothesis of improved endoscopic anastomotic detection of the UEA; however, there was a Clavien–Dindo IIIb complication in our small sample size. The operating surgeon felt that the ink contributed to the impaired tissue integrity of the bowel. This combined with the higher rate of ureteral stricture disease led the team to decide that the risks of this technique outweighed the potential benefits. Additionally, we had personal correspondence with staff at an outside institution who also halted their trial due to the accrual of safety concerns (unpublished data).

Complications are common following cystectomy and urinary diversion, with both stones and UEA strictures occurring in upwards of 11% of patients following urinary diversion [5,6,7,8,18]. These complications require access to the urinary upper tract through the UEA. Olson et al. [2] reported a 74% successful cannulation rate within the IC, with 24% of these patients requiring simultaneous percutaneous antegrade upper urinary tract access. Of the cases with no access to the ureter, 64.3% were due to a tortuous IC, 21.4% were due to a UEA stricture, and 14.2% were due to the inability to identify UEA. Hayms et al. [3] also reported a similar UEA identification success rate of 75% in IC and 33% in Indiana pouches. Initial UEA stricture management via endoscopic retrograde stent placement through the IC compared to interventional radiology management has been postulated to be more cost effective, less invasive, and involving fewer procedures [19]. In our study, we hypothesized that the ink tattooing of the UEA during urinary diversion would improve the UEA identification rates during endoscopic procedures. We also sought to evaluate the safety of ink tattooing within a urinary diversion.

Overall, ink tattooing supported the easier endoscopic identification of UEA sites. UEA were identified in 100% of the ink cohort compared to 75% of the control group. The two groups had similar overall complications rates, with the most common complications being infections. While it is not statistically significant, the ink patients had a higher UEA stricture rate compared to that of the control group (*N* = 3.30% vs. *N* = 1.9%). Additionally, to address the UEA strictures, three ink patients underwent six surgeries, including one open ureteral reimplantation, versus one control patient who a ureteral stent/percutaneous nephrostomy tube replaced to manage their UEA stricture.

The ink cohort had a higher UEA stricture rate than our prior published institutional rate of UEA stricture (6%) which was more in concert with the rates cited in the literature [5,6,7,8,18,20]. Despite our findings of a greater complications among our ink patients, this has not been the experience in other fields, specifically gastrointestinal and colorectal ones. Ink is a well-known agent used in gastrointestinal surgery to tattoo specific anatomic landmarks. Shatz et al. [14] looked at the long-term safety profile of ink tattoos in the colon in 55 tattoo sites. Ink tattoos remained in these patients for 1.5 to 117 months. No infections or pain were noted, and no endoscopic abnormalities or neoplastic changes at the tattoo sites occurred. Similarly, Shaffer et al. [15] ink tattooed the squamocolumnar junction of the esophagus and followed 19 patients endoscopically for 36 months. All of the patients who remained in the study at 36 months had good tattoo persistence without complications. Our study differs in that urine was mixed into the milieu of the bowel and an anastomosis. We postulate that urine pH has an interaction with the ink or the injection itself devascularized the bowel segment by interrupting bowel continuity; this could further create an unfavorable environment for healing. The stricture rate could be related to using ink directly adjacent to the UEA site to mark a landmark within the gastrointestinal tract. Inflammation from the ink injection, hypothetically, also could have impaired anastomosis healing.

While the number of UEA strictures was small, there was a unanimous agreement amongst the surgeons that there was a possibility that the ink had negatively impacted these patient’s outcomes. The surgeon who performed the open ureteral reimplantation hypothesized that the ink had compromised the IC bowel integrity and lead to a ureteral leak, and ultimately, the UEA stricture. Out of an abundance of caution, the trial was halted early to ensure patient safety. The surgeons reported that it was difficult to control the distribution of the ink as it was injected and that it appeared to form a bleb on the bowel wall. This may be due to using too many injections (three), too much ink, or an overly large needle. Having used the same injection protocol described in McCormick et al. [17], and as there is no other published protocol for ink injections in the setting of an IC, it is difficult to know if adjusting these factors may have improved the outcomes for our patients. If we were ever to reattempt this technique, we believe it may be reasonable to modify the protocol by using a single small dot of India Ink at the UEA, administered using a 22 gauge. However, given the outcome of the present study, it is unlikely that further attempts with India Ink would be fruitful or safe. We know of staff at another academic urology institution who also used ink to mark UEA and ended their trial due to similar concerns; unfortunately, these data have never been published. Currently, there is no published literature delineating the risks associated with the use of ink in cases of UEA in an IC. Without a method, such as the India Ink one, to help improve ureteral anastomotic visualization, surgeons are left to use the current practices. While it can be difficult and time consuming to locate a UEA endoscopically, using open procedures to address upper tract pathology is still a viable and widely used option. The development of a means to improve the identification of the ureteral anastomotic site endoscopically has the potential to reduce the procedure time and improve the patient outcomes by increasing the number of cases that can be completed endoscopically; however, this should not come with an added risk to patients on account of the technique used.

The limitations of this study include its small sample size due to the trial ending early. However, we did assess 44 renal units. As a result, our treatment and control groups differed significantly in age and in CCI score, despite randomization. Prior studies have not found age to be a predictor of urinary strictures, but they have found strictures to occur at a higher rate in males, patients with a higher BMI, and patients with grade 3 or higher Clavien–Dindo complications within 30 days of their urinary diversion [21,22]. Additionally, we chose to only evaluate the utility of ink tattooing in ileal conduits as there was concern that including other forms of urinary diversions may confound the ability to find the UEA during looposcopy and make it difficult to compare between the groups. Because of the small sample size, the study was unable to find a statistically significant difference in UEA visualization and complications between the ink and control cohorts.

## 5. Conclusions

This study showed that India Ink improved the visualization of the ureteroenteric anastomosis site following urinary diversion compared to that achieved by the normal saline control. However, the India Ink cohort experienced a higher rate of ureteroenteric strictures. The consensus among this study’s surgeons was that India Ink possibly compromised the ileal tissue integrity, and thus, the trial was stopped early out of an abundance of caution for the patient’s safety. Prudence should be used with using India Ink within urinary diversions.

## Figures and Tables

**Table 1:** Preoperative demographic data.

	India Ink (*n* = 10) *N* (%)	Control (*n* = 11) *N* (%)	*p* Value
Gender			
Male	9 (90)	8 (73)	0.59
Female	1 (10)	3 (27)	
Median Age (IQR)	72 (64, 74)	61 (58, 66)	0.04
Race			
White	9 (90)	11 (100)	0.48
Hispanic	1 (10)	0 (0)	
Median CCI (IQR)	5 (3, 7)	2 (2, 4)	0.01
Preoperative pathology			
HGPUC	8 (80)	9 (82)	1.00
CIS	0 (0)	1 (9)	
Squamous Cell	1 (10)	1 (9)	
Non Malignant	1 (10)	0 (0)	
Clinical T stage			
Tx	1 (11)	1 (9)	0.66
Ta	2 (22)	0 (0)	
Tis	0 (0)	1 (9)	
T1	2 (22)	4 (36)	
T2	4 (44)	4 (36)	
T4	0 (0)	1 (9)	
Clinical N stage			
N0	8 (89)	9 (82)	0.48
N1	1 (11)	0 (0)	
N2	0 (0)	2 (18)	
Neoadjuvant Chemotherapy			
No	5 (50)	7 (64)	0.67
Yes	5 (50)	4 (36)	
Median Preoperative GFR (IQR)	65 (47, 80)	64 (51, 79)	0.81

Interquartile Range, IQR; Charlson Comorbidity Index, CCI; High-Grade Papillary Urothelial Carcinoma, HGPUC; Carcinoma in Situ, CIS; Glomerular Filtration Rate, GFR.

**Table 2:** Surgical demographic and postoperative in-hospital complication data.

	India Ink (*n* = 10) *N* (%)	Controls (*n* = 11) *N* (%)	*p* Value
Type of Cystectomy			
Radical	8 (80)	10 (91)	0.34
Partial	2 (20)	0 (0)	
Diversion only	0 (0)	1 (9)	
Median Hospital Length of Stay (IQR)	7 (5, 12)	8 (7, 10)	0.37
Number Hospital Complications	2 (20)	5 (50)	0.34
Clavien–Dindo Score			
II	0 (0)	3 (27)	0.29
II	0 (0)	1 (9)	
IIIa	1 (10)	1 (9)	
IIIb	1 (10)	0 (0)	
Type of In Hospital Complication			
Cardiovascular (MI, stroke, DVT)	0 (0)	0 (0)	
Bowel (ileus, fistula, and small Bowel Obstruction)	1 (10)	2 (18)	
Infection (pelvis abscess, wound infection, amd sepsis)	1 (10)	0 (0)	
Kidney (pyelonephritis and ureteral stricture)	0 (0)	0 (0)	
Other	1 (10)	3 (27)	
Number Patients Discharged with Ureteral Stents	3 (30)	2 (18)	0.64
Surgical Pathology			
HGPUC	5 (50)	8 (73)	0.84
CIS	1 (10)	1 (9)	
Squamous Cell	1 (10)	0 (0)	
No Cancer	3 (30)	2 (18)	
Surgical Pathology T Stage			
T0	0 (0)	1 (9)	0.21
Ta	3 (33)	0 (0)	
Tis	1 (11)	1 (9)	
T1	0 (0)	1 (9)	
T2	3 (33)	2 (18)	
T3	1 (11)	2 (18)	
T4	1 (11)	4 (36)	
Surgical Pathology N Stage			
Nx	1 (11)	1 (9)	0.18
N0	7 (78)	5 (45)	
N1	0 (0)	0 (0)	
N2	1 (11)	5 (45)	
Median Postoperative GFR (<60 days) (IQR)	44 (24, 69)	57 (44, 69)	0.43
Median Postoperative GFR (<90 days) (IQR)	47 (24, 70)	60 (48, 72)	0.13

Interquartile Range, IQR; Myocardial Infarction, MI; Deep Venous Thrombosis, DVT; High-Grade Papillary Urothelial Carcinoma, HGPUC; Carcinoma in Situ, CIS; Glomerular Filtration Rate, GFR.

**Table 3:** Postoperative out-of-hospital complications.

	India Ink *N* (%)	Controls *N* (%)	*p* Value
Number of Patients	8 (80)	9 (82)	
Median Days Since Surgery (IQR)	51 (22, 124)	33 (14, 73)	0.28
Clavien–Dindo Classification			
1	2	3	0.16
2	11	11	
3a	7	1	
3b	2	1	
4a	0	2	
4b	0	0	
5	2	4	
Types of Complication			
Cardiovascular (MI, stroke, DVT)	1	2	
Bowel (Ileus, fistula, small bowel obstruction)	2	1	
Infection (pelvis absess, wound infection, sepsis)	14	16	
Kidney (pyelonephritis, ureteral stricture)	2	3	
Other	5	4	

Interquartile Range, IQR; Myocardial Infarction, MI; Deep Venous Thrombosis, DVT.

**Table 4:** Surgery related to ureteroenteric anastomotic stricture.

	India Ink *N* (%)	Controls *N* (%)	*p* Value
Number of Surgeries	6 (3 patients, 30%)	1 (1 patient, 9%)	0.31
Surgery Types			
PCN, Ureteral stent	4 (67)	1 (100)	
Endoscopic Dilation	1 (17)	0 (0)	
Ureteral Reimplant	1 (17)	0 (0)	
Drain Placement	0 (0)	0 (0)	

Percutaneous Nephrostomy Tube, PCN.

## Data Availability

Data are not publicly available.
